# Increasing targeting scope of adenosine base editors in mouse and rat embryos through fusion of TadA deaminase with Cas9 variants

**DOI:** 10.1007/s13238-018-0568-x

**Published:** 2018-07-31

**Authors:** Lei Yang, Xiaohui Zhang, Liren Wang, Shuming Yin, Biyun Zhu, Ling Xie, Qiuhui Duan, Huiqiong Hu, Rui Zheng, Yu Wei, Liangyue Peng, Honghui Han, Jiqin Zhang, Wenjuan Qiu, Hongquan Geng, Stefan Siwko, Xueli Zhang, Mingyao Liu, Dali Li

**Affiliations:** 1 East China Normal University and Shanghai Fengxian District Central Hospital Joint Center for Translational Medicine, Shanghai Key Laboratory of Regulatory Biology, School of Life Sciences, East China Normal University, Shanghai, 200241 China; 2 Fengxian Hospital Affiliated to Southern Medical University, Shanghai, 201499 China; 3 Xinhua Hospital, Shanghai Jiao Tong University School of Medicine, Shanghai, 200092 China; 4 School of Life Sciences, Hunan Normal University, Changsha, 410081 China; 5 Bioray Laboratories Inc., Shanghai, 200241 China; 6 Department of Molecular and Cellular Medicine, The Institute of Biosciences and Technology, Texas A&M University Health Science Center, Houston, TX 77030 USA

## Dear Editor,

The clustered regularly interspaced short palindromic repeat (CRISPR) system has been widely adapted to genome editing to either introduce or correct genetic mutations (Wang et al., 2016). However, due to competition with the dominant non-homologous end-joining (NHEJ) pathway, precise genome modifications through Cas9-stimulated homologous recombination (HR) is inefficient. Through fusion of cytidine deaminases, APOBEC1 (apolipoprotein B editing complex 1) or AID (activation-induced deaminase), with Cas9 variants, several groups have developed the cytidine base editor (BE) systems (Komor et al., 2016; Li et al., 2018; Nishida et al., 2016). The BE system achieves programmable conversion of C•G base pairs to T•A without double-stranded DNA cleavage (Zhou et al., 2017). More recently, adenine base editors (ABEs), which efficiently convert A•T base pairs to G•C in genomic DNA, have been developed via fusion of an engineered tRNA adenosine deaminase (ecTadA from *Escherichia coli*) with nCas9 (Gaudelli et al., 2017). The ABE system has quickly been adapted to generate disease models and correction of genetic disease in mice (Ryu et al., 2017; Liu et al., 2018). However, whether the editing efficiency and the targeting scope of ABE could be improved is largely unexplored. In this study, we describe the efficient generation of base-edited mice and rats modeling human diseases through ABEs with highest efficiency up to 100%. We also demonstrate an increase of ABE activity through injection of chemically modified tracrRNA and crRNA in mouse zygotes, and the expansion of editing scope by fusion of an ecTadA mutant to SaCas9n-KKH and Cas9n-VQR variants in both cells and embryos. Our study suggests that the ABE system is a powerful and convenient tool to introduce precise base conversions in rodents.

**Figure 1:**
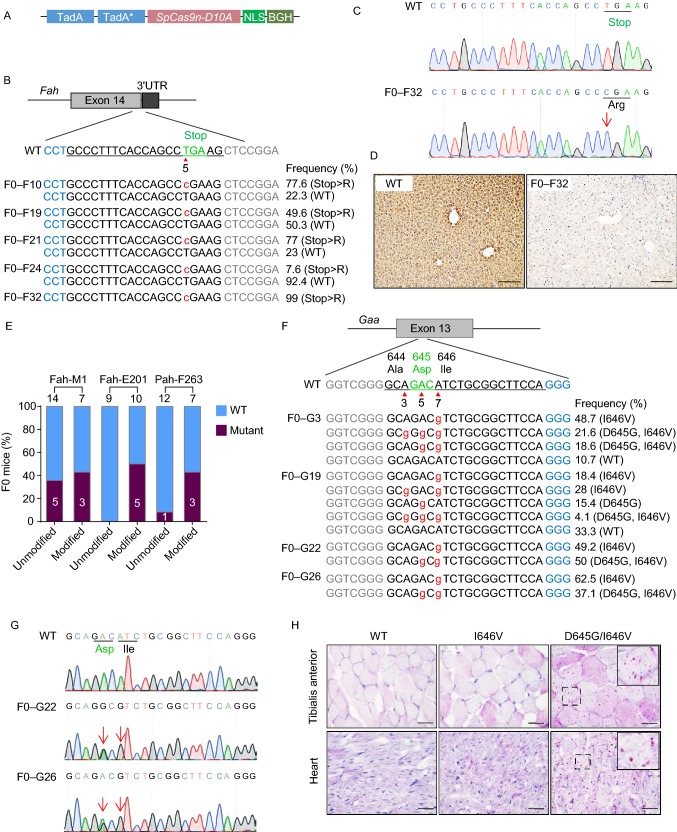
**ABE induces efficient A>G conversion in mouse and rat embryos**. (A) A schematic view of the ABE7.10 vector used as the template for ABE mRNA transcription. (B) A schematic view of the target site at the *Fah* stop codon. Target sequence is underlined. PAM sequence is labeled in blue. Stop codon is labeled in green. Arrow head indicates the targeted thymine. Base substitutions are labeled in red. Allele frequencies are listed to the right. (C) Sanger sequencing chromatograms from the WT and F0–F32 founder. T>C conversion is indicated by the red arrow. (D) IHC staining of the liver tissue sections from WT and F0–F32 founder by anti-Fah antibody. Scale bar, 100 μm. (E) The editing efficiencies at three different target sites with chemically modified crRNA/tracrRNAs or unmodified sgRNAs. The numbers indicate the number of pups generated. (F) A schematic view of the target site in exon 13 of the rat *Gaa* gene and deep sequencing results from the genomic DNA of the mutant founders. PAM sequence is labeled in blue. Target sequence is underlined with codon 644, 645 and 646 indicated by their amino acid. Base substitutions are labeled in red. Allele frequencies are listed to the right. (G) Sanger sequencing chromatograms from the genomic DNA of WT and two mutant F0 founders. Double peak signals caused by A>G conversions are indicated by red arrows. Codon 645 and 646 of WT and mutant alleles are underlined. (H) PAS staining of heart and Tibialis anterior cryo-sections from 3 week old WT, I646V and D645G/I646V homozygotes. Scale bar, 20 μm

**Figure 2:**
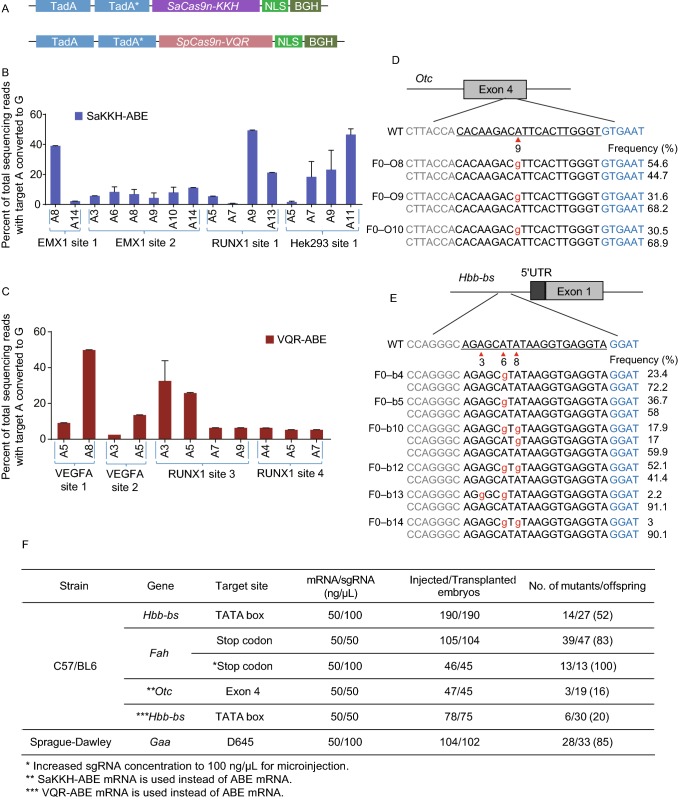
**Fusion of the adenosine deaminase with Cas9 variants**. (A) A schematic view of SaKKH-ABE and VQR-ABE vectors. (B) Frequencies of A>G conversions induced by SaKKH-ABE in HEK293T cell line. (C) Frequencies of A>G conversions induced by VQR-ABE in HEK293T cell line. (D) Genotypes of the founders produced by the SaKKH-ABE system in the *Otc* locus. Target sequence is underlined. Arrow head indicates the targeted thymine. PAM sequence is labeled in blue. Base substitutions are labeled in red. Allele frequencies are listed to the right. (E) Genotypes of the founders produced by the VQR-ABE system in the *Hbb-bs* locus. Target sequence is underlined. Arrow head indicates the targeted thymine. PAM sequence is labeled in blue. Base substitutions are labeled in red. Allele frequencies are listed to the right. (F) Summary of the targeted sites and injection parameters used to generate the point mutant rodents in the study

To test the ABE efficiency in embryos, we injected ABE mRNA (Fig. 1A) together with sgRNA targeting the TATA box of the *Hbb-bs* gene, into C57BL6 strain mouse zygotes (Fig. S1A and Table S1). Overlapping A/G peaks in the target sites were identified in 14/27 of F0 mice as determined by the chromatograms of Sanger sequencing (Figs. 2F and S1B). Further analysis by deep sequencing revealed allelic frequencies from 6%–71% among the founders (Fig. S1C). In individual allele, the editing window was extended from position A_2_–A_9_ in mouse embryos, which is broader than the window spanning position A_4_–A_7_ observed in mammalian cell lines (Gaudelli et al., 2017) (Fig. S1B and S1C). These data demonstrate that ABE is efficient to generate point mutant mice and its mutation window expands in embryos.

Next, we tested the capability of ABE to precisely mutate A:T pairs for disrupting the stop codon of the gene encoding the fumarylacetoacetate hydrolase (Fah) (Fig. 1B), whose mutations cause hereditary tyrosinemia type I (HTI) in humans. We observed high A>G conversion efficiency (39/47) among F0 mice with allelic frequencies varying from 7%–99% as determined by deep sequencing (Figs. 1B, 1C, 2F and S2A). Increasing the sgRNA concentration from 50 ng/µL to 100 ng/µL results in 100% (13/13) point mutation rate in F0 mice (Figs. 2F and S2B). Since disruption of the stop codon usually affects mRNA stability and protein expression (Frischmeyer et al., 2002), the *Fah* mRNA and protein levels were dramatically impaired (Fig. S2C and S2D). Through immunohistochemistry analysis of the liver tissue from founder F0–F32, Fah protein expression was almost undetectable (Fig. 1D) suggesting this founder was a homozygote (Fig. 1B and 1C). To investigate germline transmission efficiency, founder mice were crossed with wild type or with other founders. We observed high germline transmission efficiency (Fig. S3A and S3B). In homozygous F1 mice, the expression of *Fah* mRNA and protein was lost (Fig. S3C–E). After withdrawal of the 2-(2-nitro-4-trifluoromethylbenzoyl)-1,3-cyclohexanedione (NTBC) treatment, the phenotypes of *Fah* mutant homozygotes were similar to previous HTI model, including loss of body weight and perturbation of serum biomarkers (Shao et al., 2018) (Fig. S3F and S3G). As mutations that generate premature stop codons are common drivers in various genetic diseases (Keeling et al., 2014), ABE has a promising potential for readthrough of premature stop codons in certain genetic diseases as demonstrated in the mouse DMD model (Ryu et al., 2017).

Previous study demonstrated that 2’-O-methyl-3’-phosphorothioate (MS) modification on each ends of RNA can increase its stability, thus enhancing the Cas9 genome editing efficiency (Hendel et al., 2015). To explore whether MS modification on RNA also increases ABE activity, MS modified crRNAs and tracrRNAs were directly compared with *in vitro* transcribed sgRNAs. Indeed, in all three targets tested, the ABE editing efficiencies were higher when injected with MS modified crRNAs and tracrRNAs (Figs. 1E and S4). Moreover, the mutation efficiencies in individual mice were also increased in the group that received chemically modified RNAs (Fig. S4A–D). It suggests that increasing of sgRNA stability is an efficient strategy to increase ABE induced editing in embryos.

To investigate the activity of ABE in rats, we aimed to target the acid alpha-glucosidase (Gaa) gene to mutate aspartic acid (Asp) at codon 645 in exon 13, which is a mutation identified in glycogen storage disease type II (GSDII; Pompe disease) patients (Kroos et al., 2004) (Fig. 1F). GSDII is a fatal disorder characterized by progressive loss of skeletal and/or heart muscle function. Sanger sequencing data suggested that 85% (28/33) of rats carried single or multiple A>G substitutions between position A_3_–A_7_ in the target leading to I646V or D645G mutations (A_3_ is a synonymous mutation) (Figs. 1F, 1G and 2F). After deep sequencing of all founders, the editing frequency in individual rats ranged from 26%–100% (Fig. S5A). The founders which had higher mutation rates showed significantly reduced Gaa activity (Fig. S5B). To analyze the phenotype of the Pompe disease rat model, we crossed two pairs of founders and obtained two Gaa mutant rat strains, I646V and D645G/I646V with an overall germline transmission efficiency of 92% (12/13) (Fig. S5C). In D645G/I646V homozygous rats, Gaa enzyme activity was 0.2-0.4 nmol/h/mg which is 1.2%–2.5% of that in wild-type controls (Fig. S5D) and is similar to the enzyme activity determined in D645 mutant patients (Kroos et al., 2004). However, in I646V rats, Gaa activity was mildly reduced, suggesting that the 646 site might not be a critical site for Gaa since no point mutation at this site has been reported among 558 known mutations in patients (Pompe Mutatiedatabase http://cluster15.erasmusmc.nl/klgn/pompe/mutations.html). Gaa mutation causes abnormal accumulation of large lysosomes filled with glycogen in multiple tissues which lead to heart failure and skeletal muscle weakness depending on the severity caused by the mutation. Using PAS staining of heart, tibialis anterior and rectus femoris cryo-sections from 3 week-old *Gaa* mutant rats, we found an accumulation of PAS-positive vacuoles in all the tested tissues in D645G/I646V rats (Figs. 1H and S5E). These data suggest the successful generation of a Pompe disease rat model.

The PAM restriction of SpCas9-based ABE limits the number of potential targets. To expand the targeting scope of ABE, we fused an ecTadA variant with SaCas9n-KKH (PAM: NNNRRT) (Kleinstiver et al., 2015a) or Cas9n-VQR (PAM:NGA) (Kleinstiver et al., 2015b) to generate SaKKH-ABE and VQR-ABE respectively (Fig. 2A). To investigate the editing window and efficiency, 4 targets for either SaKKH-ABE or VQR-ABE were tested in HEK293 cells. Deep sequencing data showed that both of the ABEs actively generated A>G conversions in cells (Fig. 2B and 2C). The editing efficiencies were up to ~50% of both SaKKH-ABE and VQR-ABE variants in certain position (Fig. 2B and 2C). We noticed that the editing window of SaKKH-ABE was expanded (position A_3_–A_14_ on EMX1 site 2) compared to ABE. Our preliminary data also suggested that the highly active position of SaKKH-ABE in the target was A_8_–A_13_ which was closer to the PAM sequence compared to ABE.

To test whether these two ABEs function in mouse embryos, we injected mRNA of ABE variants with individual sgRNAs. After microinjection of SaKKH-ABE mRNA and sgRNA, 16% (3 out of 19) of the mice carried a single mutation in the *Otc* locus with an editing rate ranging from 30%–54% in single founders as determined by deep sequencing (Fig. 2D and 2F). For VQR-ABE, we also directly injected VQR-ABE mRNA and sgRNA targeting *Hbb-bs* into mouse embryos. The editing efficiency was 20% (6 out of 30) at the *Hbb-bs* locus with the A>G conversion efficiency ranging from 2%–52% as determined by deep sequencing (Fig. 2E and 2F). These data suggest that expansion of the ABE editing scope through fusion with Cas9 variants is efficient in both cell lines and mouse embryos.

To evaluate the off-target effects of this ABE, we predicted the potential off-target sites of sgRNA targeting the Fah stop codon based on sequence similarity through the on-line target prediction program (http://crispr.mit.edu/). 20 predicted off-target sites of 3 highly edited founders for each sgRNA were selected, and PCR products were amplified and subjected to deep sequencing. We found that the frequency of off-target mutation was below 0.2% (due to the threshold of Hi-Tom method) which is similar to wild-type controls by analyzing a total of 50,000–100,000 reads/site via the web site (http://www.hi-tom.net/hi-tom/), demonstrating that ABE might have very few or no off-target effects at these tested sites (Fig. S6). It suggests that ABE is an accurate base editing tools for generation of mouse and rat point mutant strains.

In summary, we demonstrated that ABE and its variants efficiently generate site-specific A:T>G:C conversions in cell lines, mouse and rat embryos. We found that the editing window of ABE7.10 in rodent embryos is from position 2–9. To the best of our knowledge, this is the first report to demonstrate efficient generation of point mutations through base editors in rats. The SaKKH-ABE and VQR-ABE system will be important tools to diversify the range of ABE targets in the genome. As A>G conversion may correct 48% of the pathogenic human SNPs (Gaudelli et al., 2017), in combination with BEs, these base editing systems have promising potential not only for generation of disease models, but more importantly for therapy of hereditary diseases caused by point substitutions.

## Electronic supplementary material

Below is the link to the electronic supplementary material.

Supplementary material 1 (PDF 906 kb)
