# Correction to: Increasing targeting scope of adenosine base editors in mouse and rat embryos through fusion of TadA deaminase with Cas9 variants

**DOI:** 10.1007/s13238-019-0631-2

**Published:** 2019-05-13

**Authors:** Lei Yang, Xiaohui Zhang, Liren Wang, Shuming Yin, Biyun Zhu, Ling Xie, Qiuhui Duan, Huiqiong Hu, Rui Zheng, Yu Wei, Liangyue Peng, Honghui Han, Jiqin Zhang, Wenjuan Qiu, Hongquan Geng, Stefan Siwko, Xueli Zhang, Mingyao Liu, Dali Li

**Affiliations:** 1 East China Normal University and Shanghai Fengxian District Central Hospital Joint Center for Translational Medicine, Shanghai Key Laboratory of Regulatory Biology, School of Life Sciences, East China Normal University, Shanghai, 200241 China; 2 Fengxian Hospital Affiliated to Southern Medical University, Shanghai, 201499 China; 3 Xinhua Hospital, Shanghai Jiao Tong University School of Medicine, Shanghai, 200092 China; 4 School of Life Sciences, Hunan Normal University, Changsha, 410081 China; 5 Bioray Laboratories Inc., Shanghai, 200241 China; 6 Department of Molecular and Cellular Medicine, The Institute of Biosciences and Technology, Texas AM University Health Science Center, Houston, TX 77030 USA

## Correction to: Protein Cell 2018, 9(9):814–819 10.1007/s13238-018-0568-x

In the original publication the grant number is incorrectly published. The correct grant number should be read as “17140901600”. The corrected contents are provided in this correction article. This work was partially supported by grants from the National Natural Science Foundation of China (Nos. 81670470 and 81600149), a grant from the Shanghai Municipal Commission for Science and Technology (17140901600, 18411953500 and 15JC1400201) and a grant from National Key Research and Development Program (2016YFC0905100).

