# Supplementary material for: Increasing targeting scope of adenosine base editors in mouse and rat embryos through fusion of TadA deaminase with Cas9 variants
Source: Protein Cell. 2018 Jul 31;9(9):814–9. doi: 10.1007/s13238-018-0568-x (PMC6107483; doi:10.1007/s13238-018-0568-x)
Supplement: Supplementary file 1 — Supplementary material 1 (PDF 906 kb) [file 13238_2018_568_MOESM1_ESM.pdf]

Materials and Methods

Construction of ABE7.10, SaKKH-ABE, VQR-ABE and sgRNA expression vectors.

Human Codon-optimized TadA-linker-TadA* sequences were codon optimized and synthesized by Idobio (Nanjing, China). PCR was performed using KOD –Plus- Neo DNA Polymerase (TOYOBO, Code:KOD-401). Fragments TadA-linker-TadA* , Cas9n(D10A) (template: Addgene Plasmid #42335), SaKKH (template: Addgene Plasmid #85170) , VQR-Cas9 (template: Addgene plasmid #101715, the clone primer was the same as Cas9n(D10A)) were amplified with primers listed in table S3. Fragments TadA-linker-TadA* and corresponding fragment Cas9n(D10A) , SaKKH , VQR-Cas9 were assembled into the pCMV-BE3 (Addgene plasmid # 73021) (digested with NotI and PmeI) using ClonExpress MultiS One Step Cloning Kit (Vazyme) to generate plasmid ABE7.10, SaKKH-ABE, VQR-ABE, respectively (Supplementary sequence 1-3). U6-sgRNA scaffold for SpCas9 or SaCas9 were amplified from PX458 or BPK2660 (Addgene Plasmid #70709) respectively, and cloned into pcDNA 3.1(+) eGFP (Addgene Plasmid #78583) to generate U6-sgRNA(sp)-EF1α-GFP or U6-sgRNA(sa)-EF1α-GFP. T7-sgRNA scaffold for SpCas9 or SaCas9 were amplified from PX458 (Addgene Plasmid #48138) or BPK2660 (Addgene Plasmid #70709), and cloned into pcDNA 3.1(+) eGFP (Addgene Plasmid #78583) to generate T7-sgRNA sp-scaffold or T7-sgRNA sa-scaffold, respectively.

Preparation of sgRNA and mRNA

The annealed sgRNAs were cloned into the T7-sgRNA sp-scaffold or T7-sgRNA sa-scaffold. The T7 promoter and different sgRNA templates were amplified using primers IVT-PCF-F/R（sp）and IVT-PCF-F/R（sa）(table S5). The sgRNAs were then transcribed using the in vitro Transcription T7 Kit (MEGAshortscript™ Kit, Ambion). The T7 promoter was introduced to ABE7.10 or SaKKH-ABE or VQR-ABE mRNA template by PCR using primers IVT-T7-ABE7.10-F/R or IVT-T7-saKKH-ABE7.10-F/R (table S5). ABE7.10, SaKKH-ABE and VQR-ABE mRNAs were transcribed using

the in vitro RNA transcription kit (mMESSAGE mMACHINE® T7 Ultra Kit, Ambion). Both sgRNA and mRNA were purified with MEGAclear™ Kit (Ambion). Chemically modified cr-RNA and trac-RNA were synthesized by Genscript (Nan jing, China) (Supplementary sequence 4).

Cell culture and transfection.

HEK293T (ATCC CRL-3216) cells lines were maintained in Dulbecco's Modified Eagle's medium (DMEM) supplemented with10% FBS (Gibico), 100 U ml^{-1} penicillin, and 100 mg ml^{-1} streptomycin at 37 °C with 5% CO2 incubation. Cells were seeded into 24-well plates (Corning) one day before transfection at a density of 200,000 cells per well, and transfected at 70–80% confluency using polyethylenimine (Polysciences) following the manufacturer's recommended protocol. For each well of a 24-well plate, a total of 500 ng DNA was used. 250 ng of SaKKH-ABE and 250 ng of sgRNA expression plasmids were transfected using 1.5 µl of polyethylenimine per well according to the manufacturer's protocol. The cells in a 24-well plate were seeded into a 12-well plate 3 days after transfection. Fluorescence-activated cell sorting (FACS) was performed 5 days after transfection following BD's recommended protocol.

Microinjection of zygotes.

For microinjection, solutions containing complexes of ABE7.10 (50 ng/µl), SaKKH-ABE (50 ng/µl) or VQR-ABE mRNA（50ng/µl） and sgRNA (50 or 100 ng/µl) were diluted in Nuclease-free Water and injected into cytoplasm using an Eppendorf transferMan NK2 micromanipulator. Injected zygotes were transferred into pseudopregnant female mice or rats immediately after injection or after overnight culture in KSOM medium at 37 °C in a humidified atmosphere consisting of 5% CO2 in air.

Animals.

C57/BL6 strain mice and Sprague-Dawley strain rats purchased from Shanghai Laboratory Animal Center were housed in standard cages in a specific pathogen-free facility on a 12 h light/dark cycle with ad libitum access to food and water. All animal

experiments conformed to the regulations drafted by the Association for Assessment and Accreditation of Laboratory Animal Care in Shanghai and were approved by the East China Normal University Center for Animal Research. To obtain homozygous mice carrying mutation on the TGA stop codon of *Fah*, the crossed founders were kept on 10 mg/L NTBC water.

Targeted deep sequencing of genomic DNA samples

HEK293T or mouse tail tip genomic DNA was isolated using the blood/cell/tissue DNA Isolation Kit (TianGen) according to the manufacturer's instructions. PCR products for targeted deep sequencing were prepared as described in HI-TOM Kits (Novogene). Mixed samples were sequenced on the Illumina HiSeq platform as previously described (www.hi-tom.net/hi-tom/documentation.html). All primers used for Hi-Tom deep sequencing are listed in table S6.

qPCR

Mice were sacrificed by carbon dioxide asphyxiation. Total RNA was extracted from mouse liver using RNAiso Plus (Takara) and reverse transcribed using HiScript II Q RT SuperMix (Vazyme). qPCRs were run on QuantStudio 3 (Applied Biosystems) using Hieff™ qPCR SYBR® Green Master Mix (YEASEN). Data were normalized to β-Actin. Primers used for qPCR are listed in table S4.

Western blot, histology and immunohistochemistry.

For western blot, mouse liver protein lysates were prepared in RIPA buffer with proteinase and phosphatase inhibitors. Proteins were separated on SDS/PAGE and transferred to nitrocellulose membranes for immunostaining with 1:500 anti-Fah antibody (AbboMax). For immunohistochemistry, mouse livers were fixed with 4% paraformaldehyde (PFA), embedded in paraffin, sectioned at 5 μm and detected with 1:2000 anti- Fah antibody (AbboMax). For measuring glycogen content, rat muscles tissues were fixed with 4% PFA, frozen in Tissue Freezing Medium (Leica), sectioned at 5 μm and stained using Glycogen Periodic Acid Schiff (PAS/Hematoxylin) Stain Kit (Solarbio).

Serum biochemical analysis.

Blood was collected using retro-orbital puncture and centrifuged at 12,000 r.p.m. at 4 ℃ for 5 min. The serum was frozen at −80°C before being sent to ADICON Clinical Laboratory to determine AST, ALT, TBIL and ALB levels.

GAA activity assay

Substrate was 6 mM 4-methylumbelliferone-alpha-glucopyranoside（Sigma）, prepared in 10% 2-methoxyethanol/0.2M phosphate citrate (PC) buffer (0.1M citric acid, 0.2 M Na2HPO4 in DI water, pH 4.3). Protein concentrations were determined by BCA Protein Assay Kit (Pierce). The acid α-glucosidase assay reaction mixture in each tube consisted of 50μL muscle tissue homogenates, 10μL 100μM acarbose, and 50μL 6mM 4-MUG. Two blank tubes with 50μL water instead of homogenates were incubated along with the samples. All tubes were incubated for 1 hour at 37°C. The reaction was stopped by addition of 1.25 mL of Glycine Carbonate buffer (glycine, 0.17 M; Na_2CO_3, 0.51M; pH 10.3). Muscle tissue homogenate (50μL) was added to each blank tube after the reaction was stopped. Fluorescence intensity was measured with a VICTOR2 spectrofluorometer (Finland, Wallac). Acid β-galactosidase at pH4.0 was used as a control. Enzyme activity is expressed as nmol/hour/mg protein.

Statistics

Data are presented as means ± DS. Means of two groups were compared using Student's t-test (unpaired, 2-tailed), with $P < 0.05$ considered to be statistically significant.

Data availability.

Plasmids encoding ABE7.10 and SaKKH-ABE are available upon request from Addgene. See Supplementary Sequences 3 for full amino acid sequences. High-throughput sequencing data have been deposited in the NCBI Sequence Read Archive database under accession code PRJNA471163.

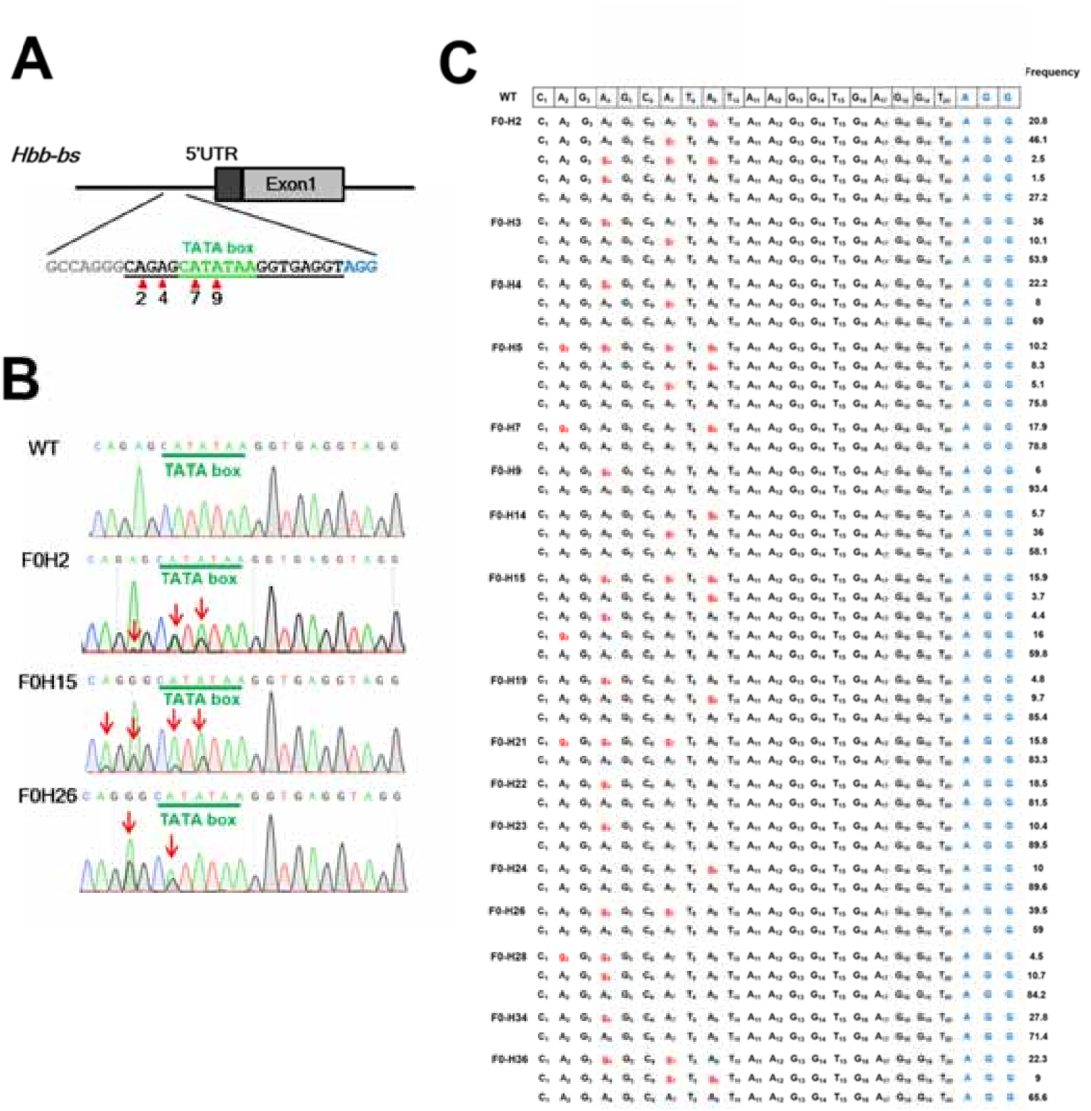

Supplementary Figure 1. Efficient A>G conversion by ABE on mouse *Hbb-bs* gene (A) schematic view of the target site in the *Hbb-bs* locus. Target sequence is underlined. PAM sequence is labeled in blue. TATA box is indicated by the green characters. Numbers represent the positions of the adenines within the editing window. (B) Sanger sequencing chromatograms of genomic DNA from WT and mutant F0 founders. Arrows indicate the double-peak signals caused by the A>G conversions (C) Deep sequencing results of the founders carrying mutations in the *Hbb-bs* locus. PAM sequences are labeled in blue. Base substitutions are labeled in red. Allele frequencies are listed to the right.

A

	Frequency (%)			Frequency (%)	
No.	T>C	WT	No.	T>C	WT
F0-F2	54.1	45.8	F0-F24	7.6	92.4
F0-F3	19.2	80.8	F0-F26	75	25
F0-F4	57.2	42.6	F0-F28	49.7	50.3
F0-F5	55.4	44.3	F0-F29	49.5	50.5
F0-F6	10.3	89.6	F0-F30	50.1	49.8
F0-F7	48.9	51	F0-F31	79.9	20.1
F0-F9	54.2	45.7	F0-F32	99	0
F0-F10	77.6	22.3	F0-F33	13.5	86.4
F0-F11	59.4	40.6	F0-F34	91.2	8.8
F0-F12	40.8	59.1	F0-F36	9.6	90.4
F0-F14	33.2	66.1	F0-F37	73	26.9
F0-F15	48.3	51.7	F0-F38	39	61
F0-F16	33.2	66.4	F0-F39	22	77.9
F0-F17	24.4	75.6	F0-F41	52.2	47.8
F0-F18	43.8	56.1	F0-F42	22.4	77.6
F0-F19	49.6	50.3	F0-F44	17.2	82.6
F0-F20	28.3	71.5	F0-F45	40.8	59.2
F0-F21	77	23	F0-F46	48.3	51.4
F0-F22	38.7	61	F0-F47	23.1	76.8
F0-F23	46	54			

B

	Frequency (%)			Frequency (%)	
No.	T>C	WT	No.	T>C	WT
F0-hF1	65.5	34.5	F0-hF8	86.2	13.8
F0-hF2	65.7	34.3	F0-hF9	62.4	37.6
F0-hF3	86.1	13.6	F0-hF10	83.4	16.6
F0-hF4	82.9	16.6	F0-hF11	62.5	37.5
F0-hF5	73.5	26.4	F0-hF12	50.2	49.8
F0-hF6	71.6	28.4	F0-hF13	72	28
F0-hF7	56.8	43.2			

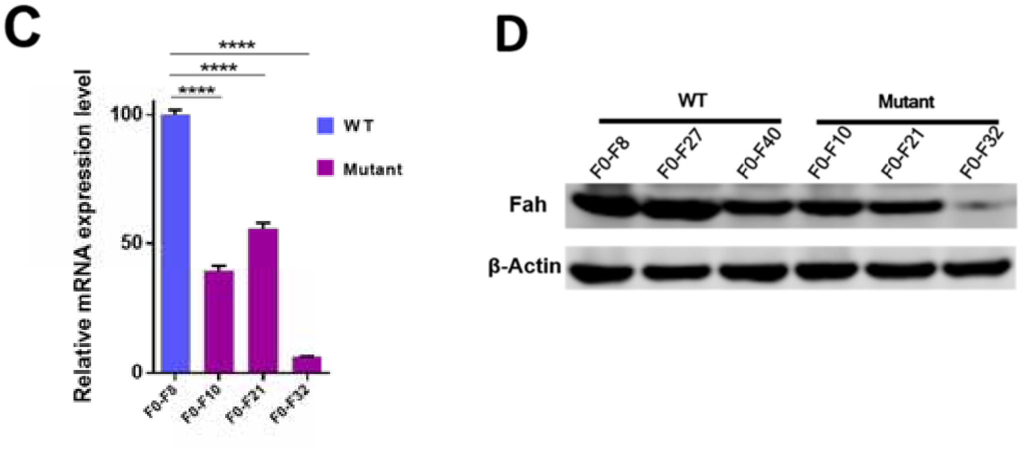

Supplementary Figure 2.Disruption of the stop condon on the mouse *Fah* gene via ABE (A-B) Allelic frequencies of each founder mutated by ABE via low sgRNA concentration (A) or high sgRNA concentration (B). (C) Relative mRNA expression of *Fah* gene from WT and three mutant F0 founders. Data presented as Mean ± SD (replicate = 3). ***, p<0.01 . (D) Western blot analysis of Fah expression in the liver tissue from the WT and mutant F0 founders.

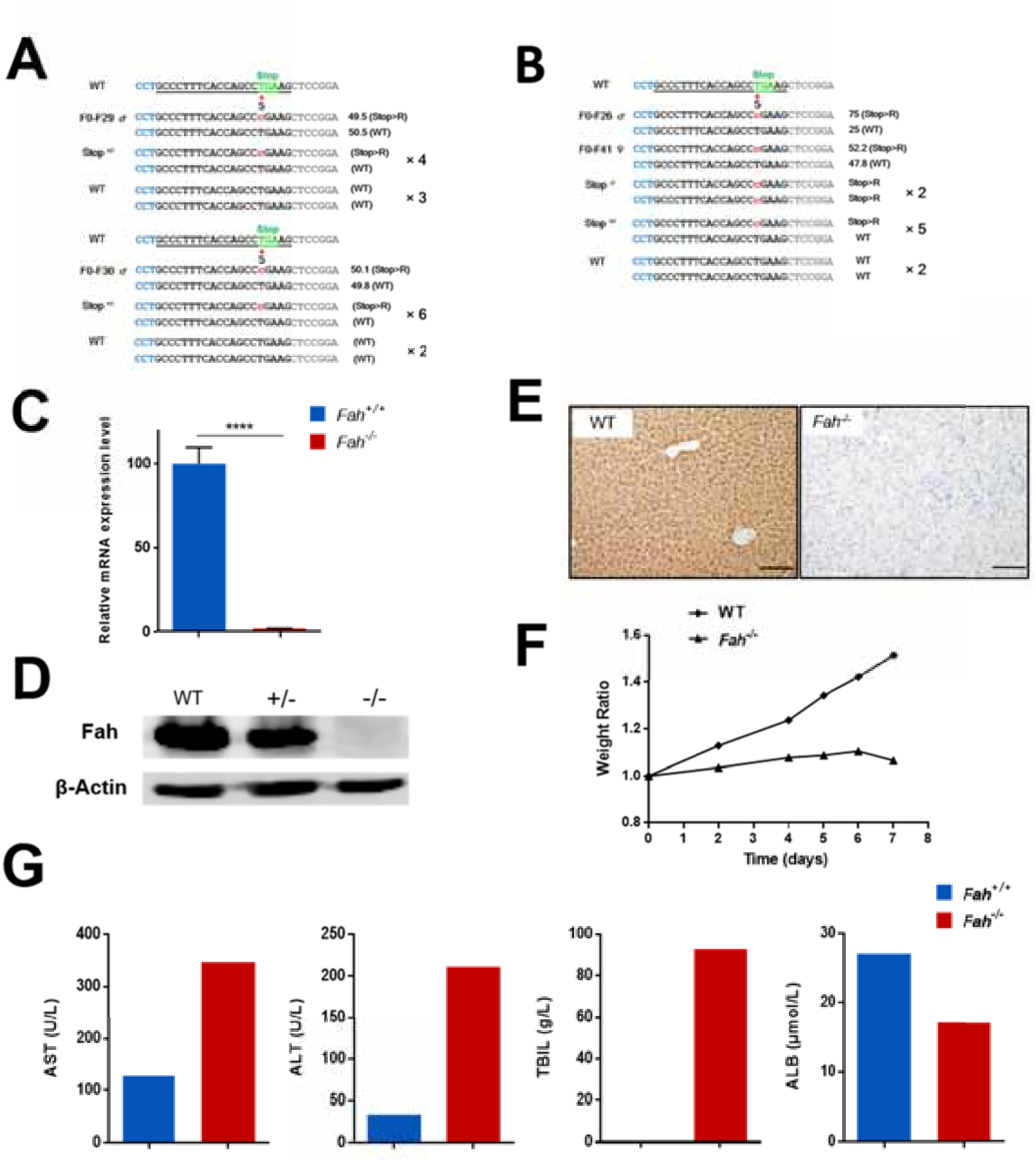

Supplementary Figure 3. Pathological defects of F1 homozygotes generated by the ABE system (A-B) Germline transmission of mutations at the Fah stop codon through crossing founders with WT mice (A) or crossing between founders (B). (C) Fah mRNA expression in Fah+/+ *and Fah-/-* mouse liver tissue. Data presented as Mean ± SD. ***, p<0.01. (D) Western blot analysis of Fah expression in the liver tissue from WT, heterozygous and homozygous offspring. (E) IHC staining of Fah protein on liver sections from WT and Fah$^{-/-}$ mouse. Scale bar, 100 μm. (F) Body weight ration of WT and Fah-/- mouse after NTBC withdrawal. (G)Serum AST, ALT, TBIL and ALB levels of WT and Fah-/- mice, 7 days after NTBC withdraw.

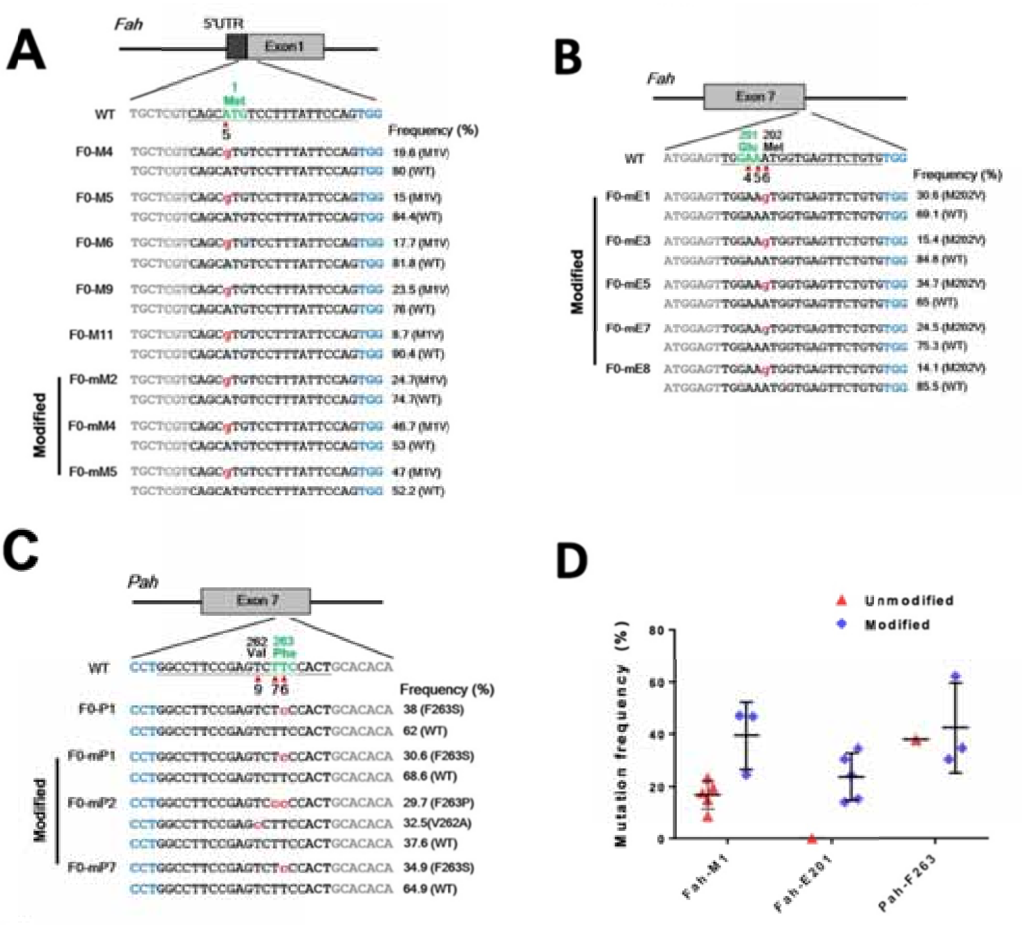

E

Strain	Gene	Target site	Methods	Dose(ng/μl)	injected/transplanted embryos	No. of mutants/offspring
C57/BL6	*Fah*	M1	mRNA/sgRNA	50/100	30/30	5/14 (35.7)
			mRNA/*crRNA/*tracRNA	50/50/50	39/37	3/7 (42.9)
		E201	mRNA/sgRNA	50/100	31/30	0/9 (0)
			mRNA/*crRNA/*tracRNA	50/50/50	29/29	5/10 (50)
	Pah	F263	mRNA/sgRNA	50/100	35/34	1/12 (8.3)
			mRNA/*crRNA/*tracRNA	50/50/50	33/31	3/7 (42.9)

* 2'-O-methyl 3'phosphorothioate (MS) modified crRNA and tracRNA is used instead of in vitro transcribed sgRNA.

Supplementary Figure 4. Increasing ABE efficiency through increasing sgRNA stability (A-C) Mutation frequencies in individual mice induced by ABE with chemically modified cr-tracRNAs in Fah-E201 site (A) , Fah-M1 site (B) and Pah-F263 site (C). PAM sequences are labeled in blue. Base substitutions are labeled in red. Allele frequencies are listed to the right. (D) The average mutation frequencies in each founder obtained via injection of modified crRNA/tracrRNAs or unmodified sgRNAs.(E) Micro-injection parameters used to inject ABE mRNA along with modified cr-tracRNAs or unmodified sgRNAs

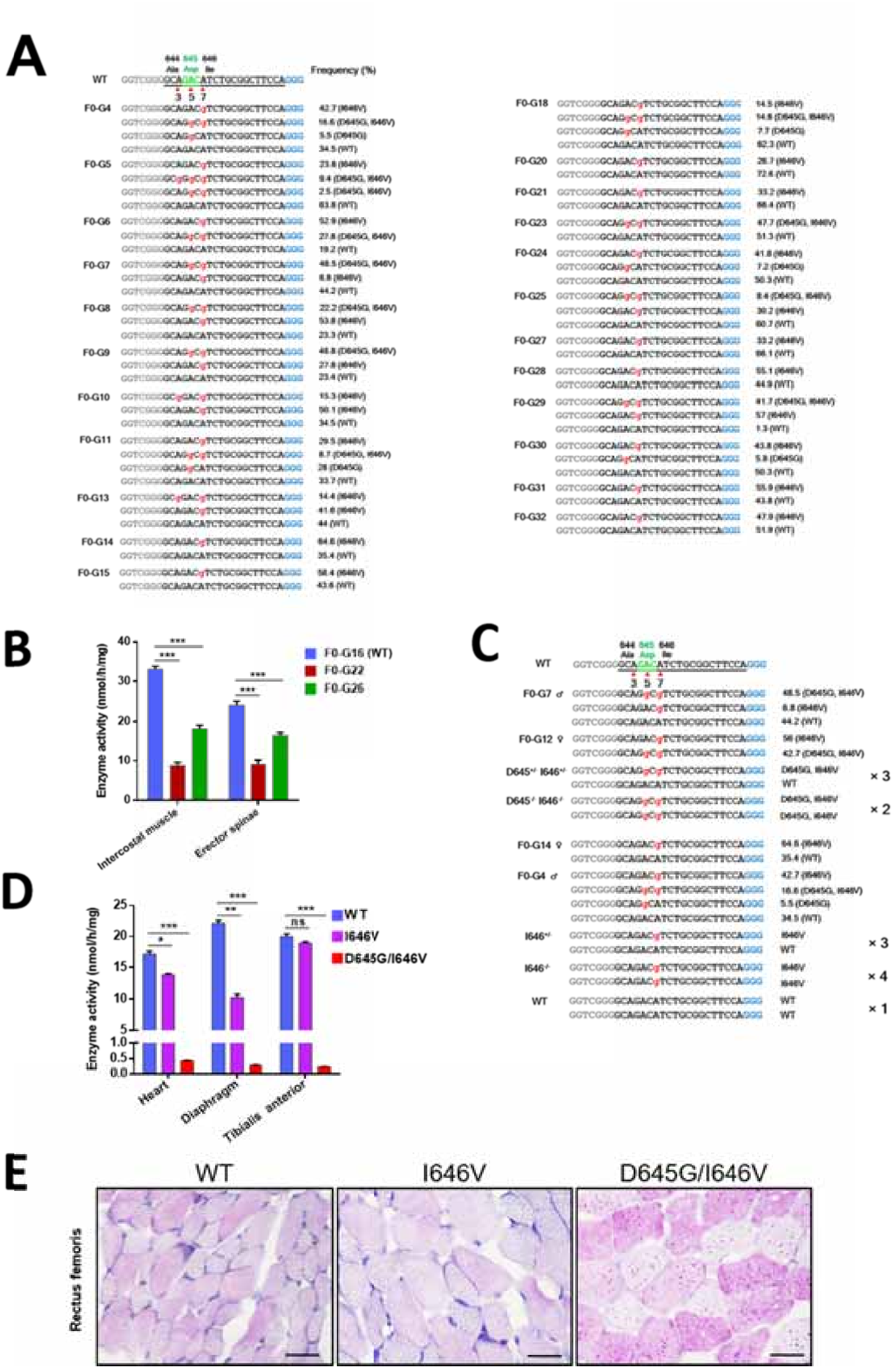
A
Frequency (%)
B
Enzyme activity (nmol/h/mg)
F0-G16 (WT)
F0-G22
F0-G26
Intercostal muscle
Erector spinae
C
D
WT
I646V
D645G/I646V
Heart
Diaphragm
Tibialis anterior
E
WT
I646V
D645G/I646V
Rectus femoris

Supplementary Figure 5. Generation of acid alpha-glucosidase mutant rats through the ABE system (A) Mutation frequencies of individual rat induced by ABE in the *Gaa* locus. PAM sequences are labeled in blue. Base substitutions are labeled in red. Allele frequencies are listed to the right. (B) Gaa enzyme activities in intercostal muscle and erector spinae from 4 week old WT and mutant founders. Data presented as Mean ±SD. (replicate = 3). ***, $p<0.01$. (C) Germline transmission of mutations at the *Gaa* locus through crossing founders with other founders. PAM sequences are labeled in blue. Base substitutions are labeled in red. F1rat quantities are listed to the right. (D) Gaa enzyme activities in heart, diaphragm, and tibialis anterior from 3 week old WT, I656V and D645G/I646V homozygous rats. Data presented as Mean ± SD. (replicate = 3). *, $P < 0.05$. ***, $P < 0.01$. (E) PAS staining of rectus femoris cyro-sections from WT, I646V and D645G/I646V homozygous rats. Scale bar, 20 μm.

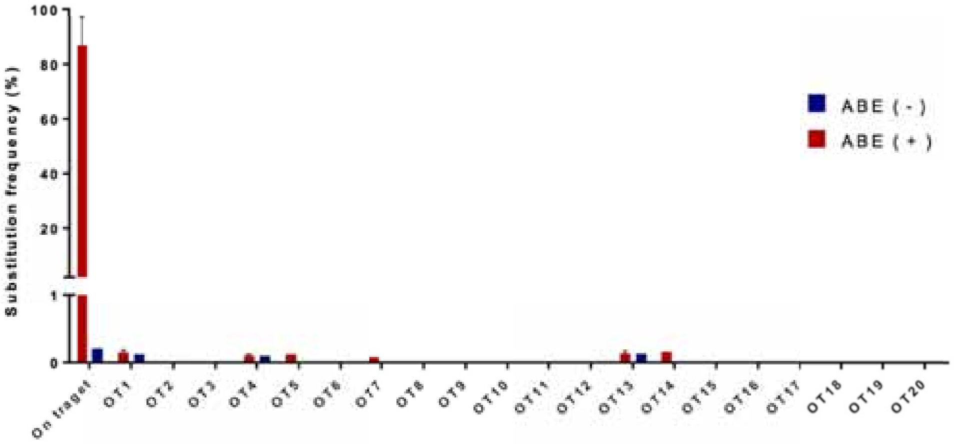

Supplementary Figure 6. Editing frequencies of ABE in predicted off target sites. Base editing frequencies of ABE at potential off target sites of Fah-stop predicted by http://crispr.mit.edu/ were measured by Hi-Tom deep sequencing. Genomic DNA from 4 week old mouse tail tips was extracted and pcr amplified for Hi-Tom deep sequencing.

Supplementary Table 1. Target sites in mouse embryos, and HEK293T cells.

		No.	Sequence (5'-3')
**ABE**	**Mouse embryos**	Fah-stop	CTTCAGGCTGGTGAAAGGGCAGG
		Fah-E201	TGGAAATGGTGAGTTCTGTGTGG
		Fah-M1	CAGCATGTCCTTTATTCCAGTGG
		Pah-F263	AGTGGAAGACTCGGAAGGCCAGG
		GAA-D645	GCAGACATCTGCGGCTTCCAGGG
		Hbb-bs	CAGAGCATATAAGGTGAGGTAGG
**SaKKH-ABE**	**HEK293T cells**	EMX1 site 1	CGGATGCACGGTCAGCGCGGGGTGGT
		EMX1 site 2	CAACCACAAACCCACGAGGGCAGAGT
		RUNX1 site 1	AAAGAGAGATGTAGGGCTAGAGGGGT
		Hek293 site 1	AGAGACACAGAGATGTCATGGAGAGT
	**Mouse embryos**	Otc	CACAAGACATTCACTTGGGTGTGAAT
**VQR-ABE**	**HEK293T cells**	VEGFA Site 1	GCGAGCAGCGTCTTCGAGAGTGAG
		VEGFA Site 2	AAACAGAGAAACTAAGGGAAAGAC
		RUNX1 site 3	AGAAAGAGAGATGTAGGGCTAGAG
		RUNX1 site 4	CAGAAGAGGGTGCATTTTCAGGAG
	**Mouse embryos**	Hbb-bs	AGAGCATATAAGGTGAGGTAGGAT

Supplementary Table 2. Potential off-target sites of Fah-stop sgRNA in the mouse genome.

No.	Sequence (5'-3')	Chromosome	Position	Direction	Mismatches
on target	CTTCAGGCTGGTGAAAGGGCAGG	chr7	91733777	-	0
OT1	CTTgAGGCTGGTaAAAGGGCTGG	chr2	110803644	+	2
OT2	CaTgAGtCTGGTGAAAGGGCTGG	chr7	124292990	+	3
OT3	CTTagGGCTGGgGAAAGGGCAGG	chr9	45541246	+	3
OT4	tTTCAGGCTGccGAAAGGGCAGG	chr15	67559328	+	3
OT5	CcTCAGGCaGGTGAAAtGGCAGG	chr19	58733308	+	3
OT6	CTTCAGGCaaGTGAAAGGaCAGG	chr18	52821719	-	3
OT7	CTTCAGtCTGaTaAAAGGGCAGG	chr7	118609893	-	3
OT8	CaTtAaGCTtGTGAAAGGGCAGG	chr9	15919356	-	4
OT9	gTcCAGGCTttTGAAAGGGCGGG	chrx	3518390	-	4
OT10	CTgggGGCTGGTGAAAtGGCAGG	chr4	62063879	+	4
OT11	ggTCAtGCTGGTGAAAtGGCTGG	chr8	9242931	-	4
OT12	CTTggGGCTaGTGAcAGGGCTGG	chr6	56312390	+	4
OT13	CTaCcGaCTGGTcAAAGGGCTGG	chr4	138147304	-	4
OT14	CTTagGtCTGGTcAAAGGGCAGG	chr4	34446483	-	4
OT15	CaTCtGGaTGGTGAAAGGGtGGG	chr15	38502392	+	4
OT16	gTcCAGGCTGcTGAAAGGGaTGG	chr16	70689741	+	4
OT17	CTaCAGcaTGGaGAAAGGGCTGG	chr2	80450019	-	4
OT18	CTTggGgCTGGgGAAAGGGCTGG	chr7	87740211	-	4
OT19	CTTtcaGCTGGaGAAAGGGCTGG	chr3	149023365	-	4
OT20	gccCAGGCTGGTGAgAGGGCTGG	chr14	67495093	-	4

Supplementary Table 3. List of primers used for plasmid construction.

Sequence ID	Sequence (5'-3')
ABE7.10-F	AGATCCGCTAGAGATCCGCGGCCGCCACCATGAGCGAGGTGGA
ABE7.10-F(in)	GAGGCTCTTCTGGAGGATCAGATAAAAAGTATTCTATTGGTTTAGCC
ABE7.10-R(in)	TGATCCTCCAGAAGAGCCTCCG
ABE7.10-R	CGAGGCTGATCAGCGGGTTTAAACTTAGACTTTCCTCTTCTTCTTGGG
saKKH-ABE-F	AGATCCGCTAGAGATCCGCGGCCGCCACCATGAGCGAGGTGGA
SaKKH-ABE-F(in)	CAGCGGAGGCTCTTCTGGAGGATCAAAAAGGAACTACATTCTGGGGCTGGCC
SaKKH-ABE-R(in)	GTTCCTTTTTGATCCTCCAGAAGAGCCTCCGCTGCTCT
SaKKH-ABE-R	ACAGTCGAGGCTGATCAGCGGGTTTAAACTTAAGCGTAATCTGGTACGTCGTATGG

Supplementary Table 4. List of primers used for q-PCR and RT-PCR.

**Sequence ID**	**Sequence (5’-3’)**
Fah-qPCR-F	CTTCGGCAGCGTGCATTC
Fah-qPCR-R	GCCATGGTATCCCACAGGTA
Mouse-Actin-F	GTGTGACGTTGACATCCGTAA
Mouse-Actin-R	CCACCGATCCACACAGAGTA

Supplementary Table 5. List of Oligonucleotides and Primers used for sgRNA and mRNA preparation.

**sequence ID**	**Sequence (5'-3')**
Fah-stop-up	TAGGCTTCAGGCTGGTGAAAGGGC
Fah-stop-dn	AAACGCCCTTTCACCAGCCTGAAG
Fah-E201-up	TAGGGTGGAAATGGTGAGTTCTGTG
Fah-E201-dn	AAACCACAGAACTCACCATTTCCAC
Fah-M1-up	TAGGCAGCATGTCCTTTATTCCAG
Fah-M1-dn	AAACCTGGAATAAAGGACATGCTG
Pah-F263-up	TAGGAGTGGAAGACTCGGAAGGCC
Pah-F263-dn	AAACGGCCTTCCGAGTCTTCCACT
GAA-D645-up	TAGGGCAGACATCTGCGGCTTCCA
GAA-D645-dn	AAACTGGAAGCCGCAGATGTCTGC
Hbb-bs-TATA-up	TAGGCAGAGCATATAAGGTGAGGT
Hbb-bs-TATA-dn	AAACACCTCACCTTATATGCTCTG
EMX1-site2-up	CACCGCAACCACAAACCCACGAGGG
EMX1-site2-dn	AAACCCCTCGTGGGTTTGTGGTTGC
EMX1-site1-up	CACCGCGGATGCACGGTCAGCGCGG
EMX1-site1-dn	AAACCCGCGCTGACCGTGCATCCGC
HEK293 site 2-up	CACCGAGAGACACAGAGATGTCATG
HEK293 site 2-dn	AAACCATGACATCTCTGTGTCTCTC
RUNX1-site1-up	CACCGAAAGAGAGATGTAGGGCTAG
RUNX1-site1-dn	AAACCTAGCCCTACATCTCTCTTTC
VEGFA-site1-up	CACCGCGAGCAGCGTCTTCGAGAG
VEGFA-site1-dn	AAACCTCTCGAAGACGCTGCTCGC
VEGFA-site2-up	CACCGAAACAGAGAAACTAAGGGAA
VEGFA-site2-dn	AAACTTCCCTTAGTTTCTCTGTTTC
RUNX1-site3-up	CACCGAGAAAGAGAGATGTAGGGCT
RUNX1-site3-dn	AAACAGCCCTACATCTCTCTTTCTC
RUNX1-site4-up	CACCGCAGAAGAGGGTGCATTTTCA
RUNX1-site4-dn	AAACTGAAAATGCACCCTCTTCTGC
Hbb-bs-up	TAGGAGAGCATATAAGGTGAGGTA
Hbb-bs-dn	AAACTACCTCACCTTATATGCTCT
Mouse-Otc- T7-up	TAGGCACAAGACATTCACTTGGGT
Mouse-Otc- T7-dn	AAACACCCAAGTGAATGTCTTGTG
IVT-PCF-F	GCGGCTTTGTTGAATAAATCGCATTCG
IVF-PCR-R(sp)	AGAGGATCCTTTAAAAGCACCGACTCGGTGCC
IVF-PCR-R(sa)	GAGGATCCAAAAAAATCTCGCCAACAAGTTGACGA
IVT-T7-ABE7.10-F	TTAATACGACTCACTATAGGGAGAGCCGCCACCATGAGCGAGGTGGA
IVT-T7-ABE7.10-R	TCCGCCTCAGAAGCCATAGA
IVT-T7-saKKH-ABE-F	TTAATACGACTCACTATAGGGAGAATGAGCGAGGTGGAGTTCAGCC
IVT-T7-saKKH-ABE-R	TCGAGGCTGATCAGCGGGTTTAAACTT

Supplementary Table 6. List of primers used for targeted deep sequencing.

**sequence ID**	**Sequence (5'-3')**
Fah-stop- hi-tom-F	GGAGTGAGTACGGTGTGCCAGTGATGTGGCTGATCCCA
Fah-stop- hi-tom-R	GAGTTGGATGCTGGATGGACTGATGCAGTGGTAGCATGA
Fah-E201- hi-tom-F	GGAGTGAGTACGGTGTGCAGCTCTGTAGCCTGGTATTGATG
Fah-E201- hi-tom-R	GAGTTGGATGCTGGATGGCCAAACAGGTGTGAAGTGCCG
Pah-F263- hi-tom-F	GGAGTGAGTACGGTGTGCCTTTCCAGCTTGTACTGGTTTC
Pah-F263- hi-tom-F-R	GAGTTGGATGCTGGATGGTTTGAGCATCCATTGTGGTTGG
Fah-M1-hi-tom-F	GGAGTGAGTACGGTGTGCTAAAGGCCCTCGGCTAGTCT
Fah-M1-hitom-R	GAGTTGGATGCTGGATGGGCTCACGTTGCTTTGAGTGG
GAA-D645- hi-tom-F	GGAGTGAGTACGGTGTGCTTGGTAACCTGGCACCACTC
GAA-D645- hi-tom-R	GAGTTGGATGCTGGATGGAGGTCATTGTGGTTCCGCAT
Hbb-bs-F	TTGTCATCACCGAAGCCTGAT
Hbb-bs-R	AAGCACCCAACTTCTTGTGAG
Hbb-bs-hi-tom-F	GGAGTGAGTACGGTGTGCGATTCCGTAGAGCCACACCC
Hbb-bs-hi-tom-R	GAGTTGGATGCTGGATGGCAGCAGCCTTCTCAGCATCA
EMX1-site2- hi-tom-F	GGAGTGAGTACGGTGTGCAAGAAGGGCTCCCATCACATCAACC
EMX1-site2- hi-tom-R	GAGTTGGATGCTGGATGGGAGTGGCCAGAGTCCAGCTTGGG
EMX1-site1- hi-tom-F	GGAGTGAGTACGGTGTGCCTTCGTGAGTGGCTTCCCTGCC
EMX1-site1- hi-tom-F	GAGTTGGATGCTGGATGGGAAGAAGGAGTGCGGGGGCTG
RUNX1-site1- hi-tom-F	GGAGTGAGTACGGTGTGCTTTAATAGGGCTTGGGGAGTCCCAG
RUNX1-site1- hi-tom-R	GAGTTGGATGCTGGATGGCATCGCTTCCTCCTGAAAATGCACC
HEK293 site2-hi-tom-F	GGAGTGAGTACGGTGTGCCTGGGTAAGGTCTACTGTGA
HEK293 site2-hi-tom-R	GAGTTGGATGCTGGATGGTCATTAAATAAATTACAAAG
VEGFA-site1-hi-tom-F	GGAGTGAGTACGGTGTGCGTGTGCAGACGGCAGTCACTAGG
VEGFA-site1-hi-tom-R	GAGTTGGATGCTGGATGGCTATTGGAATCCTGGAGTGACCCCT
VEGFA-site2-hi-tom-F	GGAGTGAGTACGGTGTGCTGAGCAATGAACCATTGGAACTTGA
VEGFA-site2-hi-tom-R	GAGTTGGATGCTGGATGGACCACTCCAGCAGAGACAACAACAT
RUNX1-site3-hi-tom-F	GGAGTGAGTACGGTGTGCTTTAATAGGGCTTGGGGAGTCCCAG
RUNX1-site3-hi-tom-R	GAGTTGGATGCTGGATGGCATCGCTTCCTCCTGAAAATGCACC
RUNX1-site4-hi-tom-F	GGAGTGAGTACGGTGTGCTTTAATAGGGCTTGGGGAGTCCCAG
RUNX1-site4-hi-tom-R	GAGTTGGATGCTGGATGGCATCGCTTCCTCCTGAAAATGCACC
Mouse-Otc- hi-tom-F	GGAGTGAGTACGGTGTGCATAGCTGGTGCAAGTACTGATGCCT
Mouse-Otc- hi-tom-R	GAGTTGGATGCTGGATGGTCTGTGAGACTTTCATTCACACCCA

Supplementary Table 7. List of primers used for deep sequencing of potential off-target sites of Fah-stop site.

sequence ID	Sequence (5’-3’)
Fah-stop-OT1-F	GGAGTGAGTACGGTGTGCTATCCTTCAACGCTCTCCTTGAT
Fah-stop-OT1-R	GAGTTGGATGCTGGATGGTTGACACTGTGACTTGTTGGCT
Fah-stop-OT2-F	GGAGTGAGTACGGTGTGCTTGCAGTGACTTGACCAACC
Fah-stop-OT2-R	GAGTTGGATGCTGGATGGTGGACATTTCTATGCCTGTGTTTAG
Fah-stop-OT3-F	GGAGTGAGTACGGTGTGCTTCCTCAGACAGGAGCAGGT
Fah-stop-OT3-R	GAGTTGGATGCTGGATGGTGCAGATCTGTGGCTGATGG
Fah-stop-OT4-F	GGAGTGAGTACGGTGTGCTCTGGCAGAAATGTTCCTCATCC
Fah-stop-OT4-R	GAGTTGGATGCTGGATGGAAAAGCATCCGTGGCTGTATC
Fah-stop-OT5-F	GGAGTGAGTACGGTGTGCCACACAGATGGAAAGGGGGA
Fah-stop-OT5-R	GAGTTGGATGCTGGATGGAGACCCCCATCCAAACCTCT
Fah-stop-OT6-F	GGAGTGAGTACGGTGTGCATGATGGTGGGCAATCCCTG
Fah-stop-OT6-R	GAGTTGGATGCTGGATGGGTATGGGTGAGGAGATGCCA
Fah-stop-OT7-F	GGAGTGAGTACGGTGTGCCATGCATCATGGTGCTAAAAGTG
Fah-stop-OT7-R	GAGTTGGATGCTGGATGGAAACTGTCCAAGATGTGACCC
Fah-stop-OT8-F	GGAGTGAGTACGGTGTGCATGCTCCCCAACTGGGTCAA
Fah-stop-OT8-R	GAGTTGGATGCTGGATGGTATTTAGCACGTCCTATGGGCA
Fah-stop-OT9-F	GGAGTGAGTACGGTGTGCCAAAGGCAAATGACCTGGACC
Fah-stop-OT9-R	GAGTTGGATGCTGGATGGAAGCCTGAATTTGCGCGG
Fah-stop-OT10-F	GGAGTGAGTACGGTGTGCACTCCTCAATGTCGTCGAGC
Fah-stop-OT10-R	GAGTTGGATGCTGGATGGACTGTTCCTGTGTACAGATTCAT
Fah-stop-OT11-F	GGAGTGAGTACGGTGTGCAGGGTTAGTCTCAAAGAGGGGT
Fah-stop-OT11-R	GAGTTGGATGCTGGATGGTGATACTCAGATCTGCTACAAGAGA
Fah-stop-OT12-F	GGAGTGAGTACGGTGTGCCAAGCCCTGCTGACATGCAA
Fah-stop-OT12-R	GAGTTGGATGCTGGATGGCGGGCAGCCATCATTGTAGG
Fah-stop-OT13-F	GGAGTGAGTACGGTGTGCGGACACTGGAGGCCCTTCTAT
Fah-stop-OT13-R	GAGTTGGATGCTGGATGGATCCCACTGGGGACAATGATAC
Fah-stop-OT14-F	GGAGTGAGTACGGTGTGCCCAGGGATCTCAAAAATCTAGCC
Fah-stop-OT14-R	GAGTTGGATGCTGGATGGTAAGGCACTGCAGGTAACTTCC
Fah-Stop-OT15-F	GGAGTGAGTACGGTGTGCATGAGGGTGTACCTTAGAGAGGC
Fah-stop-OT15-R	GAGTTGGATGCTGGATGGAGGATGGCGTCAGCAGCAAA
Fah-Stop-OT16-F	GGAGTGAGTACGGTGTGCTCTGAGTGTTCCTTTCAGCCATA
Fah-stop-OT16-R	GAGTTGGATGCTGGATGGCATGAAGGCATGCTAAGTGAAAC
Fah-Stop-OT17-F	GGAGTGAGTACGGTGTGCACCCAGCTCCATTGCTTTCA
Fah-stop-OT17-R	GAGTTGGATGCTGGATGGTCCTAACGGGGTAACCAAAGC
Fah-Stop-OT18-F	GGAGTGAGTACGGTGTGCGGGGGTTTTTCTCTGTGATTTGC

Fah-stop-OT18-R	GAGTTGGATGCTGGATGGTAGCAGGTTTGTGATGTTTATGC
Fah-Stop-OT19-F	GGAGTGAGTACGGTGTGCGAGGGTGTGTGGCTTCAACTG
Fah-stop-OT19-R	GAGTTGGATGCTGGATGGTCTTGCAAAAGTTTAAGAAGAGCCT
Fah-Stop-OT20-F	GGAGTGAGTACGGTGTGCCCTTCTAGATCGCAGCCTCTT
Fah-stop-OT20-R	GAGTTGGATGCTGGATGGTGCTAGAGTAGGGCCAAGAAC

Supplementary Sequences 1. Amino acid sequences of ABE.
MSEVEFSHEYWMRHALTLAKRAWDEREVPVGAVLVHNNRVIGEGWN-
RPIGRHDPTAHAEIMALRQGGLVMQNYRLIDATLYVTLEPCVMCAGAMIHSRIGRVVF-
GARDAKTGAAGSLMDVLHHPGMNHRVEITEGILADECAALLSDFFRMRRQEIKAQK-
KAQSSTDSGGSSGGSSGSETPGTSESATPESSGGSSGGSSEVEFSHEYWMRHALTLAKRAR-
DEREVPVGAVLVLNNRVIGEGWNRAIGLHDPTAHAEIMALRQGGLVMQNYRLIDATLYVT-
FEPCVMCAGAMIHSRIGRVVFGVRNAKTGAAGSLMDVLHYPGMNHRVEITEGILADECAALL-
CYFFRMPRQVFNAQKKAQSSTDSGGSSGGSSGSETPGTSESATPESSGGSSGGSDKKYSIG-
LAIGTNSVGWAVITDEYKVPSKKFKVLGNTDRHSIKKNLIGALLFDSGETAEATRL-
KRTARRRYTRRKNRICYLQEIFSNEMAKVDDSFFHRLEESFLVEEDKKHERHPIFGNI-
VDEVAYHEKYPTIYHLRKKLVDSTDKADLRLIYL-
ALAHMIKFRGHFLIEGDLNPDNSDVDKLFIQLVQTYNQLFEENPINASGVDAKAIL-
SARLSKSRRLENLIAQLPGEKKNGLFGNLIALSLGLTPNFK-
SNFDLAEDAKLQLSKDTYDDDLDNLLAQIGDQYADLFLAAKNLSDAILLSDILRVN-
TEITKAPLSASMIKRYDEHHQDLTLLKALVRQQLPEKYKEIFFDQSKNGYAGYIDG-
GASQEEFYKFIKPILEKMDGTEELLVKLNREDLLRKQRTFDNGSIPHQIHLGELHAIL-
RRQEDFYPFLKDNREKIEKILTFRIPYYVGPLARGNSRFAWMTRKSEETIT-
PWNFEEVVDKGASAQSFIERMTNFDKNLPNEKVLPKHSLLYEYFTVYNELTKVKYVTEGMRK-
PAFLSGEQKKAIVDLLFKTNRKVTVKQLKEDYFKKIECFDSVEISGVEDRF-
NASLGTYHDLLKIIKDKDFLDNEENEDILEDIVLTLTLFEDREMI-
EERLKTYAHLFDDKVMKQLKRRRYTGWGRLSRKLINGIRDKQSGKTILDFLKSDGFANRN-
FMQLIHDDSLTFKEDIQKAQVSGQGDSLHEHIANLAGSPAIKKGILQTVKVVDELVKVMGRHK-
PENIVIEMARENQTTQKGQKNSRERMKRIEEGIKELGSQILKE-
HPVENTQLQNEKLYLYYLQNGRDMYVDQELD-
INRLSDYDVDHIVPQSFLKDDSIDNKVLTRSDKNRGKSDNVPSEEVVKKMKNY-
WRQLLNAKLITQRKFDNLTKAERGGLSELDKAGFIKRQLVETRQITKHVAQILDSRM-
NTKYDENDKLIREVKVITLKSKLVSDFRKDFQFYKVREINNYHHAHDAYLNAVVGTALIK-
KYPKLESEFVYGDYKVYDVRKMIAKSEQEIGKATAKYFFYSNIMNFFKTEITLANGEIRKR-
PLIETNGETGEIVWDKGRDFATVRKVLSMPQVNIVKKTEVQTGGF-
SKESILPKRNSDKLIARKKDWDPKKYGGFDSPTVAYSVLVVAKVEKGK-
SKKLKSVKELLGITIMERSSFEKNPIDFLEAKGYKEVKKDLIIKLPKYSLFELENGRKRMLA-
SAGELQKGNELALPSKYVNFLYLASHYEKLKG-
SPEDNEQKQLFVEQHKHYLDEIIEQISEFSKRVILADANLDKVLSAYNKHRDKPIREQAE-
NIIHLFTLTNLGAPAAFKYFDTTIDRKRYTSTKEVLDATLIHQSITGLYETRIDLSQLGG-
DSGGSPKKKRKV*

Supplementary Sequences 2. Amino acid sequences of SaKKH-ABE.

MSEVEFSHEYWMRHALTLAKRAWDEREVPVGAVLVHNNRVIGEGWN-
RPIGRHDPTAHAEIMALRQGGLVMQNYRLIDATLYVTLEPCVMCAGAMIHSRIGRVVF-
GARDAKTGAAGSLMDVLHHPGMNHRVEITEGILADECAALLSDFFRMRRQEIKAQK-
KAQSSTDSGGSSGGSSGSETPGTSESATPESSGGSSGGSSEVEFSHEYWMRHALTLAKRAR-
DEREVPVGAVLVLNNRVIGEGWNRAIGLHDPTAHAEIMALRQGGLVMQNYRLIDATLYVT-
FEPCVMCAGAMIHSRIGRVVFGVRNAKTGAAGSLMDVLHYPGMNHRVE-
ITEGILADECAALLCYFFRMPRQVFNAQKKAQSSTDSGGSSGGSSGS-
ETPGTSESATPESSGGSSGGSKRNYILGLAIGITSVGYGI-
IDYETRDVIDAGVRLFKEANVENNEGRRSKRGARRL-
KRRRRHRIQRVKKLLFDYNLLTDHSELSGINPYEARVKGLSQKLSEEEFSAALL-
HLAKRRGVHNVNEVEEDTGNELSTKEQISRNSKALEEKYVAELQLERLKKDGEVRG-
SINRFKTSDYVKEAKQLLKVQKAYHQLDQSFIDTYIDLLETRRTYYEGPGEG-
SPFGWKDIKEWYEMLMGHCTYFPEELRSVKYAYNADLYNALNDLNNL-
VITRDENEKLEYYEKFQIIENVFKQKKKPTLKQIAKEILVNEEDIKGYRVTSTGKPEFT-
NLKVYHDIKDITARKEIIENAELLDQIA-
KILTIYQSSEDIQEELTNLNSELTQEEIEQISNLKGYTGTHNLSLKAINLILDELWHTND-
NQIAIFNRLKLVPKKVDLSQQKEIPTTLVDDFILSPVVKRSFIQSIKVINAIIK-
KYGLPNDIIIELAREKNSKDAQKMINEMQKRNRQTNERIEEIIRTTGKENAKYLIEKI-
KLHDMQEGKCLYSLEAIPLEDLLNNPFNYEVDHIIPRSVSFDNSFNN-
KVLVKQEENSKKGNRTPFQYLSSSDSKISYETFKKHILNLAKGKGRIS-
KTKKEYLLEERDINRFSVQKDFINRNLVDTRYATRGLMNLLRSYFRVNNLDVKVKS-
INGGFTSFLRRKWKFKKERNKGYKHHAEDALIIANAD-
FIFKEWKKLDKAKKVMENQMFEEKQAESMPEIETEQEYKEIFITPHQIKHIK-
DFKDYKYSHRVDKKPNRKLINDTLYSTRKDDKGNTLIVNNLNGLYDKDNDKLKKLINK-
SPEKLLMYHHDPQTYQKLKLIMEQYG-
DEKNPLYKYYEETGNYLTKYSKKDNGPVIKKIKYYGNKLNAHLDITDDYPNSRN-
KVVKLSLKPYRFDVYLDNGVYKFVTVKNLDVIKKENYYEVNSKCYEEAKKLKKISNQAE-
FIASFYKNDLIKINGELYRVIGVNNDLLNRIEVNMIDITYREYLENMNDKRPPHIIK-
TIASKTQSIKKYSTDILGNLYEVKSKKHPQIIKKGGSPKKKRKVSSYPYDVPDYA*

Supplementary Sequences 3. Amino acid sequences of VQR-ABE.
MSEVEFSHEYWMRHALTLAKRAWDEREVPVGAVLVHNNRVIGEGWN-
RPIGRHDPTAHAEIMALRQGGLVMQNYRLIDATLYVTLEPCVMCAGAMIHSRIGRVVF-
GARDAKTGAAGSLMDVLHHPGMNHRVEITEGILADECAALLSDFFRMRRQEIKAQK-
KAQSSTDSGGSSGGSSGSETPGTSESATPESSGGSSGGSSEVEFSHEYWMRHALTLAKRAR-
DEREVPVGAVLVLNNRVIGEGWNRAIGLHDPTAHAEIMALRQGGLVMQNYRLIDATLYVT-
FEPCVMCAGAMIHSRIGRVVFGVRNAKTGAAGSLMDVLHYPGMNHRVE-
ITEGILADECAALLCYFFRMPRQVFNAQKKAQSSTDSGGSSGGSSGS-
ETPGTSESATPESSGGSSGGSDKKYSIG-
LAIGTNSVGWAVITDEYKVPSKKFKVLGNTDRHSIKKNLIGALLFDSGETAEATRL-
KRTARRRYTRRKNRICYLQEIFSNEMAKVDDSFFHRLEESFLVEEDKKHERHPIFGNI-
VDEVAYHEKYPTIYHLRKKLVDSTDKADLRLIYL-
ALAHMIKFRGHFLIEGDLNPDNSDVDKLFIQLVQTYNQLFEENPINASGVDAKAIL-
SARLSKSRRLENLIAQLPGEKKNGLFGNLIALSLGLTPNFK-
SNFDLAEDAKLQLSKDTYDDDLDNLLAQIGDQYADLFLAAKNLSDAILLSDILRVN-
TEITKAPLSASMIKRYDEHHQDLTLLKALVRQQLPEKYKEIFFDQSKNGYAGYIDG-
GASQEEFYKFIKPILEKMDGTEELLVKLNREDLLRKQRTFDNGSIPHQIHLGELHAIL-
RRQEDFYPFLKDNREKIEKILTFRIPYYVGPLARGNSRFAWMTRKSEETIT-
PWNFEEVVDKGASAQSFIERMTNFDKNLPNEKVLPKHSLLYEYFTVYNELT-
KVKYVTEGMRKPAFLSGEQKKAIVDLLFKTNRKVTVKQLKEDYFKKIECFDSVEIS-
GVEDRFNASLGTYHDLLKIIKDKDFLDNEENEDILEDIVLTLTLFEDREMI-
EERLKTYAHLFDDKVMKQLKRRRYTGWGRLSRKLINGIRDKQSGKTILDFLKSDGFANRN-
FMQLIHDDSLTFKEDIQKAQVSGQGDSLHEHIANLAGSPAIK-
KGILQTVKVVDELVKVMGRHKPENIVIEMARENQTTQKGQKNSRERMKRIEE-
GIKELGSQILKEHPVENTQLQNEKLYLYYLQNGRDMYVDQELD-
INRLSDYDVDHIVPQSFLKDDSIDNKVLTRSDKNRGKSDNVPSEEVVKKMKNY-
WRQLLNAKLITQRKFDNLTKAERGGLSELDKAGFIKRQLVETRQITKHVAQILDSRM-
NTKYDENDKLIREVKVITLKSKLVSDFRKDFQFYKVREINNYHHAHDAYLNAVVGTALIK-
KYPKLESEFVYGDYKVYDVRKMIAKSEQEIGKATAKYFFYSNIMNFFKTEITLANGEIRKR-
PLIETNGETGEIVWDKGRDFATVRKVLSMPQVNIVKKTEVQTGGF-
SKESILPKRNSDKLIARKKDWDPKKYGGFVSPTVAYSVLVVAKVEKGK-
SKKLKSVKELLGITIMERSSFEKNPIDFLEAKGYKEVKKDLIIKLPKYSLFELENGRKRMLA-
SAGELQKGNELALPSKYVNFLYLASHYEKLKG-
SPEDNEQKQLFVEQHKHYLDEIIEQISEFSKRVILADANLDKVLSAYNKHRDKPIREQAE-
NIIHLFTLTNLGAPAAFKYFDTTIDRKQYRSTKEVLDATLIHQSITGLYETRIDLSQLGG-
DSGGSPKKKRKV*

Supplementary Sequences 4. Chemically modified crRNA and tracrRNA used in this study. The 2′-*O*-methyl 3′phosphorothioate (MS) modified bases are colored *red* and marked with *.

Fah-E201-crRNA
U*G*G*AAAUGGUGAGUUCUGUGGUUUUAGAGCUAUGCUGUUUUG

Pah-F263-crRNA
A*G*U*GGAAGACUCGGAAGGCCGUUUUAGAGCUAUGCUGUUUUG

Fah-M1-crRNA
C*A*G*CAUGUCCUUUAUUCCAGGUUUUAGAGCUAUGCUGUUUUG
tracrRNA
ACAGCAUAGCAAGUUAAAAUAAGGCUAGUCCGUUAUCAACUUGAAAAAGUGGCAC-
CGAGUCGGUGC**U*U*U***U
